# An Analytical Compendium of the Various Branches of Medical Science, for the Use and Examination of Students

**Published:** 1852-01

**Authors:** 


*An Analytical Compendium of the various branches of Medical Science, for the use and examination of Students.* By JOHN NEILL, M. D., Demonstrator of Anatomy in the University of Pennsylvania, etc., and FRANCIS GURNEY SMITH, M. D., Lecturer on Physiology in the Philadelphia Association for Medical Instruction, etc. *Second Edition, revised and improved.* Philadelphia, Blanchard & Lea, 1852.

We notice with much pleasure the early call for a second edition of this useful work. The necessity for *compendiums* and *manuals*, for the benefit of students, particularly while in attendance upon lectures, when systematic study of voluminous text-books is impossible, has been long felt and admitted; and numerous publications of the kind have appeared both in Europe and this country. Of the volume before us, we can scarcely allow ourselves to speak in the terms which its great merit deserves; but its value and popularity have been attested by the rapid exhaustion of the first edition, published only three years since. The present edition has been carefully revised and improved, and in part re-written. It should be in the hands of every student in our medical colleges. B.

